# Recruiting underrepresented individuals in a double pandemic: Lessons learned in a randomized control trial

**DOI:** 10.1017/cts.2021.843

**Published:** 2021-08-25

**Authors:** Gretchen E. White, Chelsea N. Proulx, Natalia E. Morone, Audrey J. Murrell, Doris M. Rubio

**Affiliations:** 1 Institute for Clinical Research Education, University of Pittsburgh Schools of the Health Sciences, Pittsburgh, PA, USA; 2 General Internal Medicine, Boston University School of Medicine, Boston, MA, USA; 3 Boston Medical Cente, Boston, MA, USA; 4 College of Business Administration, University of Pittsburgh, Pittsburgh, PA, USA

**Keywords:** COVID-19, diversity equity inclusion, anti-racism, career development, diversifying the biomedical research workforce, recruitment

## Abstract

The Building Up Trial is a cluster-randomized trial that aims to address the issue of the leaky career pathway for underrepresented (UR) faculty in biomedical fields. Regulatory approval and recruitment for the Building Up Trial took place during the COVID-19 pandemic and the anti-racism movement. The pandemic and anti-racism movement personally and professionally impacted the target population and made recruitment challenging at both the institution and participant level. The target sample size for this study was 208 postdoctoral fellows or early-career faculty across 26 predominately white institutions. Challenges and adaptations are described. The Building Up Trial was delayed by 3 months. In total, 225 participants from 26 institutions were enrolled. Participants are predominately female (80%), Hispanic/Latinx (34%) or non-Hispanic/Latinx Black (33%), and early-career faculty (53%). At the institution level, obtaining Institutional Review Board (IRB) approval through a single Institutional Review Board (sIRB) posed the biggest challenge. We adapted to COVID-19-related challenges through simplifying sIRB forms, modifying study practices, and increasing communication with institutions. Recruiting UR postdoctoral fellows and faculty during the COVID-19 pandemic and anti-racism movement was challenging but not impossible. Studies should be prepared to modify study and recruitment policies to overcome additional barriers posed by the pandemics.

## Introduction

The lack of racial and ethnic diversity in the biomedical research workforce and the fact that underrepresented (UR) individuals disproportionately leave biomedical research is well-documented [1–3]. Despite increases in the number of scientists from UR backgrounds in biomedical sciences, those from UR backgrounds leave faculty positions at an alarming rate [4,5]. This may be because they encounter more deterrents than those from majority backgrounds as they attempt to progress through their career [6]. To address this issue for UR [1–3] faculty in biomedical fields, the Building Up a Diverse Workforce for Biomedical Research (Building Up) Trial aims to test the effectiveness of an intervention [7] designed for postdoctoral fellows and junior faculty who are UR in health-related sciences [8].

Regulatory approval and recruitment for the Building Up Trial took place during the COVID-19 pandemic, which has upended research in health-related sciences [9,10]. During this time, postdoctoral fellows have experienced suspension of their research activities, strained relationships with supervisors, and worry about their job prospects [11]. Junior faculty members have also been hit hard^10,12^ as they juggle online teaching, clinical work, family caretaking, and remote schooling [13–15]. Women faculty have experienced greater negative impact on their research activities, which is especially problematic for women of color [16]. Those who are UR in the biomedical research field, and therefore eligible to participate in the Building Up Trial, are disproportionately affected by the pandemic.

This is compounded by the current anti-racism movement, which increased awareness and communication surrounding issues of structural racism, hate crimes, police brutality, and disparities. As a result, the “minority tax,” which describes the disproportionate burden on UR faculty to participate in diversity efforts,[17,18] has multiplied as institutions look to UR faculty for help in developing anti-racism policies and practices. Because these services are usually uncompensated, the minority tax compounds the effort of sustaining academic careers in the biomedical sciences at a time when many junior research investigators, particularly UR faculty, are leaving their research careers [4,5].

The combination of the COVID-19 pandemic and the anti-racism movement have simultaneously made recruitment for research studies more challenging and emphasized the importance of programs aimed at curbing the departure of UR faculty from biomedical research [19]. Therefore, the purpose of this report is to describe recruitment challenges and lessons learned from recruiting UR postdoctoral fellows and junior faculty during the COVID-19 pandemic and anti-racism movement, as they pertain to the Building Up Trial.

## Methods

### Study Design

Building Up is a cluster-randomized trial at 26 institutions. The trial compares two interventions lasting 10 months and including four components (i.e., mentoring, monthly sessions, networking, and coursework). In Intervention A, participants are assigned a near-peer mentor at their institution, whom they meet with monthly and as needed. Near-peer mentors are mid-career faculty members who can lend first-hand insight, advice, and support to those slightly junior to them. The near-peer mentor also organizes networking opportunities for participants at their institution. Near-peer mentors were directly recruited by site champions. To prevent perpetuating the minority tax, Building Up covered percent effort for all near-peers and their administrative staff. Additionally, participants in Intervention A complete coursework in grant writing and medical writing. Participants in Intervention B continue usual mentoring, networking, and coursework as needed offered at their institutions. Both Interventions A and B attend a monthly Excellence in Leadership Webinar series. Due to the nature of the intervention, site champions and participants were not blinded to their intervention assignment.

### Participants

To be eligible to participate in the trial, participants must (1) be from an UR background (see Supplemental Table 1 for definition of UR in health-related sciences) [8], (2) have a terminal degree (MD, PhD, PharmD, etc.), (3) be a postdoctoral fellow or early career faculty within the first 6 years of appointment, (4) be committed to a career in clinical, basic, or translational research, and (5) have approximately 50% protected research time. To enroll in the study, participants were required to complete the informed consent process and apply to the study using an application modeled after that used in the original intervention [20]. Initially, the application required a letter of support from the director of the participant’s postdoctoral program, division chief, department chair, or dean guaranteeing 50% protected research time.

The target sample size for this study was 208 participants, or 8−10 participants at 26 predominately white institutions.

### Measures

In the application to the program, we asked participants to describe why they were eligible for the study. First participants were asked to “specify your gender,” “select one or more race that you identify with,” “are you of Hispanic or Latino ethnicity (meaning a person of Cuban, Mexican, Puerto Rican, South or Central American or other Spanish culture or origin, regardless of race)?” To determine whether someone was from an UR background, we also asked the following: “Building Up aims to jump-start the careers of underrepresented junior investigators by providing them with the mentoring, skills, and knowledge needed for successful research careers." The NIH defines "underrepresented in biomedical research" under the following link: https://grants.nih.gov/grants/guide/notice-files/NOT-OD-20-031.html. Please write a statement as to why you qualify for this study that is geared toward people who are underrepresented.” This information was used to determine whether someone was from a disadvantaged background since we did not ask about it separately. At preintervention research assessments, completed in July−September 2020, participants reported their age, gender, race and ethnicity, career status, disability status, whether they were raised by adults with a bachelor’s degree, and their scientific discipline.

### Recruitment Methods

Recruitment for the Building Up Trial occurred at two levels; at the institution level, and once the institution had IRB approval, at the participant level. First, we recruited institutions to be a site for the trial, which required finding a champion at each site. Second, the champion recruited participants for the trial.

### Data Analysis

Summary statistics are reported. We analyzed data using SAS version 9.4 (SAS Institute, Cary, NC, USA).

## Results

### Study Participants

There were 225 participants from 26 institutions enrolled in the Building Up Trial. Participants in the Building Up Trial are predominately female (80%), Hispanic/Latinx (34%) or non-Hispanic/Latinx Black (33%), faculty (53%), and raised by adults with a bachelor’s degree (62%) (Table 1).

**Table 1.** – *Characteristics of under-represented post-doctoral fellows and early career faculty in the Building Up a Diverse Workforce for Biomedical Research Trial*

Characteristic	*n*=225^a^	(%)^b^
Age (median, 25th−75th percentile)	36	33−40
Gender		
Male	43	(19.6)
Female	175	(79.9)
Gender minority	1	(0.5)
Race/ethnicity		
Hispanic/Latinx	75	(34.1)
Non-Hispanic/Latinx	145	(65.9)
White	29	(13.2)
Black	73	(33.2)
Other	29	(13.2)
Multi-race	14	(6.4)
Career status		
Post-doctoral fellow	102	(46.6)
Faculty	117	(53.4)
Disability		
Yes	11	(5.5)
No	190	(84.4)
Raised by adults with a bachelor’s degree		
Yes	136	(61.8)
No	84	(38.2)

^a^ Numbers may not add to total because of missing values.

^b^ Unless otherwise specified.

### Challenges with Institution Recruitment

**Fig. 1.**
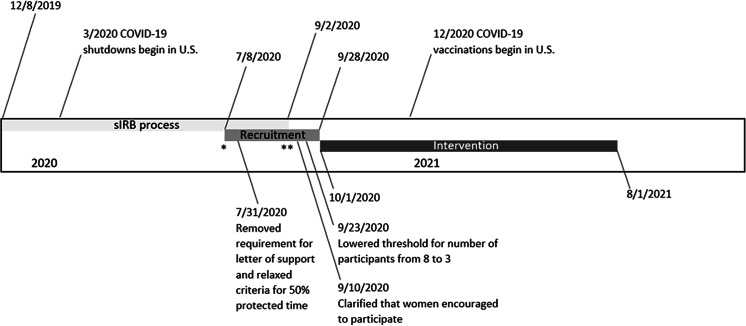
Building Up a Diverse Workforce for Biomedical Research Trial Timeline. * Initial anticipated intervention start date July 1, 2020. ** Second anticipated intervention start date September 1, 2020. sIRB, single Institutional Review Board.

At the institution level, obtaining IRB approval through a single institutional review board (sIRB) posed the biggest challenge. Because gaining sIRB approval took an unanticipated length of time, it required that we delay our targeted study start date by 2 months (Fig. 1).

The amount of review and information requested from institution IRBs was highly variable. Some institution IRBs required a detailed review at their home institution prior to ceding review to the University of Pittsburgh while others quickly ceded review. One institutional IRB required an IRB modification after ceding review to the University of Pittsburgh which delayed recruitment at that institution for 7 weeks. Additionally, frequent errors in returned forms often required correction and new signatures from institution IRB representatives, delaying approval. In response to these common errors, the University of Pittsburgh began providing partially completed forms to institutions needing approval. With standardized information prefilled (e.g., role of the institution, institution-specific recruitment procedures, participant payment, etc.), delays due to errors were reduced.

Institutions reported to the University of Pittsburgh Building Up Team that the COVID-19 pandemic further impacted the IRB approval process. First, in mid-March 2020, institution IRBs began fast-tracking review for COVID-19-related research and some institutions reported a short-term suspension of review for studies unrelated to the pandemic. Second, in early pandemic hotspots, clinical and administrative obligations hindered site champions from completing required IRB paperwork and submissions. Third, IRB staff were required to work from home and did not always have access to technology needed to sign and/or scan documents. It took a median of 94 days (25th−75th percentile: 63−220 days) for 26 institutions (Supplemental Table 2) to be onboarded by the University of Pittsburgh’s IRB. Seventy-seven percent of institutions began the sIRB approval process in December 2019, well before the COVID-19 pandemic began (Table 2). But less than half of these 20 institutions were onboarded by the time the pandemic hit the USA.

**Table 2.** Single institutional review board approval timeline for the Building Up Trial

Institution	sIRB initiated	Onboarded	Duration (days)
A	12/8/2019	6/19/2020	194
B	12/8/2019	1/28/2020	51
C	12/8/2019	1/28/2020	51
D	12/8/2019	1/28/2020	51
E	12/8/2019	2/11/2020	65
F	12/8/2019	6/19/2020	194
G	12/8/2019	2/11/2020	65
H	5/19/2020	6/19/2020	31
I	12/8/2019	2/11/2020	65
J	12/8/2019	2/11/2020	65
K	12/8/2019	6/19/2020	194
L	12/8/2019	1/28/2020	51
M^a^	12/8/2019	2/11/2020	65
N	12/8/2019	7/15/2020	220
O	5/19/2020	7/15/2020	57
P	12/8/2019	7/15/2020	220
Q	6/19/2020	7/15/2020	26
R	12/8/2019	7/15/2020	220
S	12/8/2019	7/15/2020	220
T	5/14/2020	7/15/2020	62
U	12/11/2019	8/4/2020	237
V	12/8/2019	8/4/2020	240
W	12/8/2019	8/13/2020	249
X	5/19/2020	9/2/2020	106
Y	3/19/2020	8/27/2020	161
Z	12/8/2019	8/4/2020	240
AA^b^	Not initiated	Not onboarded	NA
AB^c^	2/6/2020	Not onboarded	NA
AC^d^	Not initiated	Not onboarded	NA
AD^e^	Not initiated	Not onboarded	NA
AE^f^	4/21/2020	Not onboarded	NA
AF^g^	Not initiated	Not onboarded	NA
Median (25th−75th percentile) 94 (63−220) days			

NA, not applicable; sIRB, single Institutional Review Board.

^a^Institution M excluded from the study due to low recruitment on September 29, 2020.

^b^Institution AA: Site declined to participate in January 2020.

^c^Institution AB: Site declined to participate in February 2020.

^d^Institution AC: Lost site for other reason (i.e., participating in similar research study) in May 2020.

^e^Institution AD: Site declined to participate in June 2020.

^f^Institution AE: Site declined to participate in June 2020.

^g^Institution AF: Lost site for other reason (i.e., delayed substantially because of COVID) in June 2020.

Due to a variety of factors (i.e., not obtaining IRB approval, being clinically overwhelmed by the COVID-19 pandemic), seven institutions dropped out of the study (Supplemental Fig. 1). One of these institutions was onboarded by the University of Pittsburgh IRB but excluded from the study on September 29, 2020, after recruitment had concluded, because they were not successful in recruiting participants.

### Challenges with Participant Recruitment

**Fig. 2.**
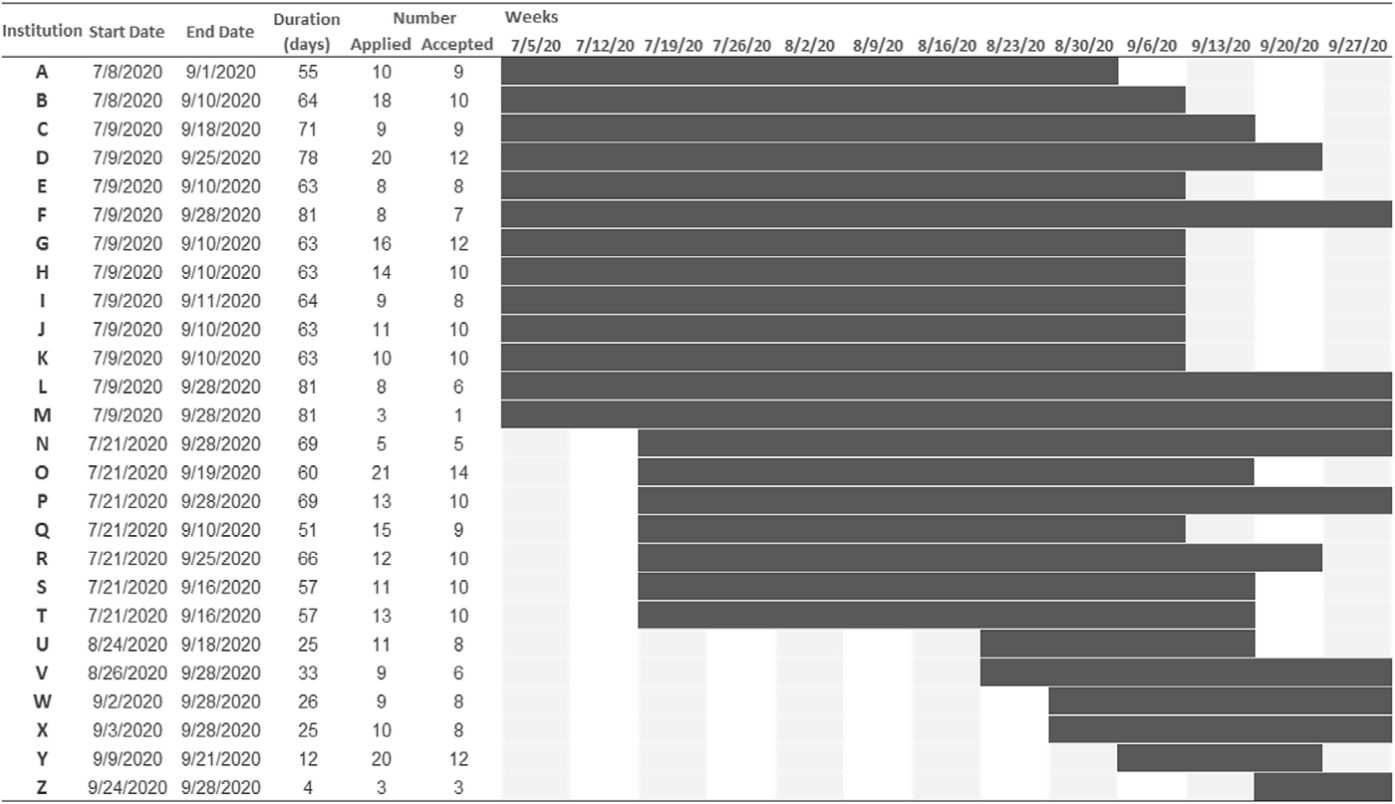
Recruitment timeline for institutions participating in the Building Up Trial.

In July 2020, the 20 institutions onboarded by the University of Pittsburgh IRB were asked to begin recruiting participants (Fig. 2). The other six institutions began recruitment immediately upon onboarding in August or September 2020. Recruitment was primarily conducted by the site champion at each institution. Some site champions also asked near-peer mentors to aid in recruitment. The number of applications received and accepted was highly variable by institution (Fig. 2; range: 3−21 people applied and 1−14 people accepted). In general, recruitment was slow (median: 63 days; 25th−75th percentile: 52−68 days) and delayed our second target study start date from September to October 2020 (Fig. 1). To promote participant-level recruitment, we modified recruitment-related study policies and increased communication with institutions.

### Modified Study Policies

As of July 31, 2020, 4 weeks before our second target study start date, we had only received 34 completed applications across the 22 onboarded institutions. At this time, we removed the requirement for the letter of support, as it posed a barrier for completing the application. We also, because COVID-19 had altered clinical demands and state, local, and university policies restricted research activities, relaxed the criteria for 50% protected research time.

On September 10, 2020, we clarified with institutions that the NIH encourages women to participate in programs designed for recruitment, retention, and career development [8]. After this clarification, many site champions began using nontargeted recruitment techniques such as listservs to recruit women and recruitment improved considerably. Of the 51 female participants who were not from racial or ethnic groups UR in health-related sciences [8], 96% had a disability, were from disadvantaged backgrounds, and/or worked in scientific disciplines where women are UR [21]. On September 23, 2020, because some institutions were struggling to recruit 8−10 participants and because the prior success of the intervention at the University of Pittsburgh with 3−4 participants, we lowered the threshold for the number of participants at each institution from 8 to 3. To achieve our target sample size, we compensated for institutions with fewer participants by enrolling up to 14 participants at institutions with more recruitment success.

### Increased Communication

We increased communication with institutions and site champions. First, we provided weekly updates to site champions to inform them of the number of participants that had applied and been accepted. This feedback allowed site champions to quickly alter recruitment practices based on what was and was not working. These updates also provided opportunities for bidirectional communication between the University of Pittsburgh and site champions. We communicated frequently with site champions through both email and videoconferencing, as needed. Second, we distributed a newsletter celebrating recruitment progress and informing institutions of modified study policies. Third, we quickly communicated recruitment success stories and answers to recruitment questions to all institutions. Fourth, a University of Pittsburgh principal investigator contacted leadership at Diversity and Inclusion and/or Faculty Development offices at each institution that was struggling with recruitment since, anecdotally, involving more people in recruitment efforts seemed to improve recruitment numbers.

## Discussion

We found that recruiting UR postdoctoral fellows and faculty during the COVID-19 pandemic and anti-racism movement was challenging but it is possible to successfully recruit during these times. Institutions participating in this research study were diverse and recruitment challenges during the COVID-19 pandemic were often unique to each institution, but there were important lessons learned. First, we should have allocated more time to the sIRB approval process as it was much more time consuming than we anticipated. While the purpose of an sIRB is to streamline approval so it can proceed as quickly as possible [22], we found that it took a median of 94 days for institutions to be onboarded by the University of Pittsburgh IRB. This is significantly longer than what has been reported in other studies (9−81 days)[23–25] but likely shorter than approval time without an sIRB[25]. The time to sIRB onboarding may be partially explained by the University of Pittsburgh IRB not using tools created to streamline the sIRB process, such as the online Smart IRB portal and the IREx system, during the approval process for this study. These tools may help prevent delays in the future. Other delays may be explained by changes to IRB policies as the research landscape shifted during the COVID-19 pandemic. Consistent with our experiences, research has shown that IRB offices were prioritizing reviews of COVID-related research and changes in protocols for research that was shifting from in-person to online [26]. However, it should be noted that not all delays were caused by the COVID-19 pandemic as 9 of the 21 (43%) institutions that began the sIRB process prior to the onset of the COVID-19 pandemic in the USA were onboarded to the sIRB before March 2020. We were also surprised that some institution IRBs administered detailed reviews rather than abbreviated reviews prior to or even after ceding to a single IRB. However, this is consistent with other studies and researchers should anticipate variations in local IRB willingness to cede to an sIRB and resulting differences in review type and length [27].

Second, networking through the Clinical and Translational Science Award (CTSA) Consortium proved invaluable. When institutions dropped out of the study, we were able to capitalize on relationships with colleagues at CTSAs and quickly recruit new institutions. In fact, at least one institution reached out to a trial principal investigator to request participation in the trial. Moreover, during the recruitment period, many potential participants were comfortable reaching out to site champions and study staff at the University of Pittsburgh to let us know that they were personally and professionally impacted by both the COVID-19 pandemic and the anti-racism movement. Being aware of and responsive to these challenges allowed us, as the lead institution, to identify issues early and modify plans and communication strategies accordingly. Many of the communication strategies that we employed, such as partially completing IRB-related forms; regularly meeting with study personnel to discuss progress and troubleshoot challenges; distributing biweekly newsletters to provide study updates, highlight success stories, and troubleshoot challenges; and disseminating lessons learned back to all site teams have been identified as novel approaches to site engagement and may provide useful in future multisite trials [28].

Third, we found that involving more people in recruitment, such as near-peer mentors and those in Diversity and Inclusion and Faculty Development offices was fruitful and should have been done earlier in the process. In addition, the role of near-peer mentors was important and peer meeting is a positive source of support especially for UR scholars within health sciences [29]. Despite the role of the near-peer mentor, recruitment did not differ by intervention allocation. Institutions that engaged in nontargeted recruitment methods, such as sending recruitment materials via listservs or departmental emails, had more applications because self-identification is often the only way to identify UR postdoctoral fellows and faculty when using the NIH definition. This is contrary to what has been published in the literature for recruiting UR participants into clinical trials [30,31].

This study is subject to the following limitations. First, we did not assess the reason for the variability in recruitment success by site champions nor did we collect organizational characteristics for the participating institutions. Second, we did not collect information from site champions on how many postdoctoral fellows or junior faculty members they approached to participate in the study. Moreover, because information is not readily available for most institutions, we do not know how many postdoctoral fellows and junior faculty at each institution were eligible to participate. This may have impacted recruitment success at institutions that struggled. Third, expanded eligibility may have impacted our original study question. We encouraged women in all biomedical research fields to apply for the program, without preference for the specific field in which they worked. Despite this, all but two women in the program met the definition for UR in biomedical science because of their racial or ethnic identity, disability status, disadvantaged background, or work in a scientific field where women are UR. We also relaxed the criteria that all participants engage in research at least half the time. However, during a time when many had increased clinical duties due to COVID-19 surges, we believed that it would be irresponsible to ignore the impact that COVID-19 had on participants’ research careers. We reviewed every application to ensure that participants were dedicated to a research career. Furthermore, we did not compare institutions with low versus high recruitment success because only six institutions did not successfully meet our original recruitment target of eight participants and only one institution did not meet our revised recruitment target of three participants. Finally, the application asked participants to list why they were eligible to participate but we did not ask them to list every reason they were eligible to participate. Therefore, it is possible that the two women who did not disclose a disability, a disadvantaged background, and/or work in scientific disciplines where women are UR, actually had or did one of these things.

Based on our experience, we recommend the following for investigators conducting similar research. First, nontargeted recruitment approaches such as using listservs may improve recruitment and should be considered. We found that the benefit of additional participants self-identifying as UR outweighed the cost of the additional time study staff spent screening participants for eligibility. Second, we suggest including Diversity and Inclusion and/or Faculty Development offices early in the process as they can aid in recruitment. Third, while the sIRB should improve efficiencies in the approval process, we found that precompleting forms was effective in reducing errors and recommend others to do the same.

## Conclusions

Because of our ability to adapt to the unprecedented environment, we were able to begin the Building Up Trial within 3 months of our targeted start date and exceed our recruitment goal by 17 participants. Recruiting during the COVID-19 pandemic and anti-racism movement was challenging but not impossible. Studies should be prepared to modify study and recruitment policies to overcome additional barriers posed by the pandemic.

## Supplementary material

For supplementary material accompanying this paper visit http://dx.doi.org/10.1017/cts.2021.843. click here to view supplementary material
