# Supplementary material for: Recruiting underrepresented individuals in a double pandemic: Lessons learned in a randomized control trial
Source: J Clin Transl Sci. 2021 Aug 25;5(1):e185. doi: 10.1017/cts.2021.843 (PMC8596076; doi:10.1017/cts.2021.843)
Supplement: Supplementary file 1 [file S2059866121008438sup001.docx]

**Recruiting Under-represented Individuals in a Double Pandemic, Lessons Learned in a Randomized Control Trial**

**Supplementary Material**

### Supplemental Table 1. Underrepresented Populations in the U.S. Biomedical, Clinical, Behavioral and Social Sciences Research Enterprise

| Individuals from racial and ethnic groups^a^ |
| --- |
| Blacks or African Americans, Hispanics or Latinos, American Indians or Alaska Natives, Native Hawaiians, and other Pacific Islanders |
| Individuals with disabilities |
| Those with a physical or mental impairment that substantially limits one or more major life activities, as described in the Americans with Disabilities Act of 1990, as amended. |
| Individuals from disadvantaged backgrounds |
| Meet two or more of the following:  - Were or currently are homeless, as defined by the McKinney-Vento Homeless Assistance Act - Were in the foster care system, as defined by the Administration for Children and Families - Were eligible for the Federal Free and Reduced Lunch Program for two or more years - Have/had no parents or legal guardians who completed a bachelor’s degree - Were or currently are eligible for Federal Pell grants - Received support from the Special Supplemental Nutrition Program for Women, Infants and Children (WIC) as a parent or child  Grew up in one of the following areas: a) a U.S. rural area, as designated by the Health Resources and Services Administration (HRSA) or b) a Centers for Medicare and Medicaid Services-designated Low-Income and Health Professional Shortage Areas |
| Women^b^ |

### This table is created based on information from: National Institutes of Health Office of the Director Scientific Workforce Diversity. Underrepresented Populations in the U.S. Biomedical, Clinical, Behavioral and Social Sciences Research Enterprise. December 3, 2020, 2020. Updated February 7, 2020. Accessed April 19, 2021. <https://diversity.nih.gov/about-us/population-underrepresented>

### ^a^Underrepresentation can vary from setting to setting; individuals from racial or ethnic groups that can be demonstrated convincingly to be underrepresented by the grantee institution should be encouraged to participate in NIH programs to enhance diversity.

^b^NIH encourages institutions to consider women for faculty-level, diversity-targeted programs to address faculty recruitment, appointment, retention or advancement

**Supplemental Table 2**. Institutions Onboarded by the University of Pittsburgh Institutional Review Board

| Albert Einstein College of Medicine/Montefiore Medical Center |
| --- |
| Children’s National Medical Center/The George Washington University |
| East Carolina University |
| Loyola University Medical Center |
| Massachusetts General Hospital |
| Mayo Clinic Rochester |
| Medical University of South Carolina |
| Northwestern University |
| Oregon Health and Science University |
| Penn State Health |
| Rush University Medical Center |
| Texas A&M University |
| Tufts University Health Sciences |
| University of Buffalo |
| University of California, Davis |
| University of Chicago |
| University of Colorado Denver Anschutz Medical Campus |
| University of Michigan |
| University of Minnesota |
| University of Pennsylvania Perelman School of Medicine |
| University of Southern California |
| University of Texas Health Science Center at San Antonio/University of Texas at Austin |
| University of Virginia |
| University of Wisconsin, Madison |
| Vanderbilt University Medical Center |
| Washington University at St. Louis School of Medicine |

**Supplemental Figure 1**. Institution and Participant Flow Diagram for the Building Up Trial


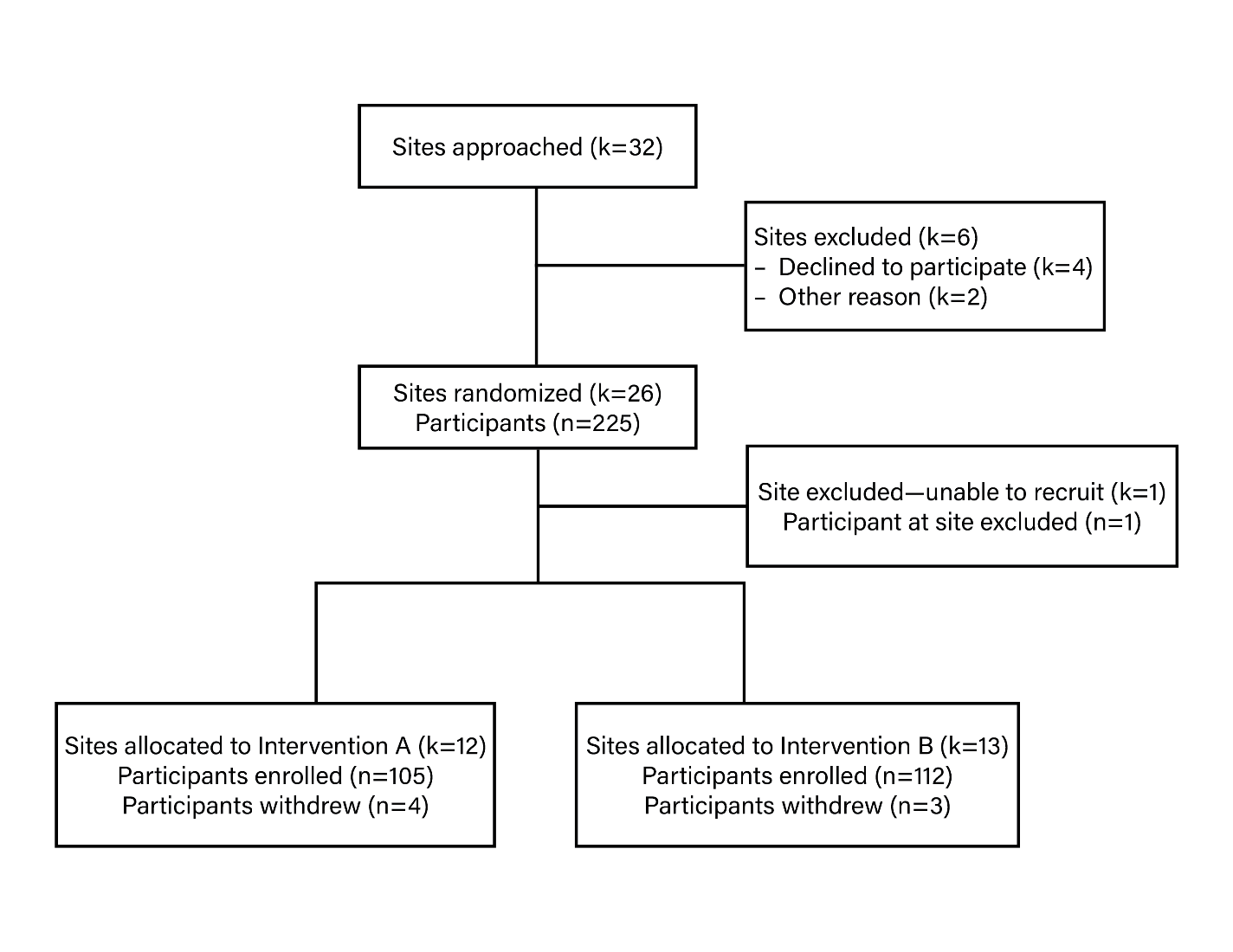


^a^

^b^

^c^

^a^ Sites declined to participate: Institution AA, January 2020; Institution AB, February 2020; Institution AD, June 2020; Institution AE, June 2020.

^b^ Sites excluded for other reason: Institution AC (i.e., participating in similar research study), May 2020; Institution AF (i.e., delayed substantially because of COVID), June 2020.

^c^ Site excluded for low recruitment: Institution M, September 29, 2020.
